# Comparative analysis of NSG and NBSGW mice for preclinical evaluation of gene-modified human hematopoietic stem and progenitor cells

**DOI:** 10.1186/s13287-026-05010-8

**Published:** 2026-04-15

**Authors:** Caroline Y. Kuo, Sean Harrington, Beatriz Campo-Fernandez, Stacia K. Wyman, Xiaomeng Wu, Ruixue Zhang, Alejandro Espinoza, Bruno J. de Andrade Silva, Julie M. Sanchez, Sorel Fitz-Gibbon, Chin-hong Tseng, Matteo Pellegrini, Melissa Bonner, Zulema Romero

**Affiliations:** 1 Division of Allergy and Immunology, Department of Pediatrics, David Geffen School of Medicine, University of California, Los Angeles, CA USA; 2 Formerly of Genetix Biotherapeutics, Somerville, MA USA; 3 Department of Microbiology, Immunology and Molecular Genetics, David Geffen School of Medicine at the University of California, Los Angeles, CA USA; 4 Innovative Genomics Institute, University of California, Berkeley, CA USA; 5 Department of Molecular Cell and Developmental Biology, University of California, Los Angeles, CA USA; 6 Division of Dermatology, Department of Medicine, David Geffen School of Medicine at the University of California, Los Angeles, CA USA; 7 Department of Medicine, School of Medicine, University of California, Los Angeles, CA USA

**Keywords:** Humanized mouse models, Hematopoietic stem and progenitor cells (HSPCs), Long-term engraftment, Lentiviral vector (LVV), CRISPR/Cas9

## Abstract

Humanized mouse models are essential for evaluating the engraftment capacity and genetic integrity of gene-modified hematopoietic stem and progenitor cells (HSPCs). Here, we compared two widely used xenotransplantation platforms, NSG and NBSGW mice, in the context of lentiviral vector (LVV) transduction and CRISPR/Cas9-mediated gene correction. HSPCs harboring high LVV copy numbers exhibited engraftment deficits in NSG mice that were not observed in NBSGW mice. This discrepancy highlights the potential for the NBSGW model to mask safety liabilities of LVV-modified products due to its higher overall levels of human chimerism. In contrast, CRISPR/Cas9 editing with a single-stranded oligodeoxynucleotide donor yielded comparable correction rates in both models, even across decreasing input cell doses, demonstrating that long-term repopulating hematopoietic stem cells (HSCs) retain equivalent engraftment capacity in each strain. Single-cell RNA-sequencing revealed distinct progenitor populations that were markedly under-represented in the NSG model but preserved in NBSGW recipients, emphasizing the greater capacity of NBSGW mice to better support multilineage human hematopoiesis. Together, these findings establish that both NSG and NBSGW mice are suitable for assessing long-term engraftment and gene modification outcomes in human HSPCs. However, the significantly higher percentage of human cell chimerism in the NBSGW model may obscure cell populations with engraftment deficits. Careful selection of in vivo models is therefore critical for rigorous preclinical evaluation of gene therapy products prior to clinical translation.

**Supplementary Information:**

The online version contains supplementary material available at 10.1186/s13287-026-05010-8.

## Introduction

Humanized mouse models are critical preclinical platforms for assessing the long-term engraftment of human hematopoietic stem and progenitor cells (HSPCs) and evaluating the durability of genome modification in vivo. The NSG (NOD-*Prkdc*^*scid*^*Il2rg*^*null*^) mouse strain harbors mutations in the *Prkdc* and *IL2rg* genes on the NOD genetic background, resulting in a complete absence of adaptative immunity and severe impairments in innate immune function. The immunodeficient background of these models permits engraftment of human HSPCs following cytoreductive irradiation [1], although this approach can be associated with significant toxicity. Alternatively, busulfan-based conditioning has been used [2], but generally requires higher doses of CD34^+^ cells to ensure long term engraftment [3]. Thus, both conditioning regimens have inherent limitations, underscoring the need for alternative models that enable efficient human HSPC engraftment.

Introduction of a hypomorphic, loss of function c-kit mutation (*Kit*^*W41/W41*^) into the NSG background gave rise to the NBSGW mouse model, enabling durable human engraftment without the need for cytoreductive conditioning. *C-kit* encodes a tyrosine kinase receptor (KIT) expressed on hematopoietic cells, melanocytes and certain neural and germ cells [4], and is essential for normal hematopoiesis. NBSGW mice transplanted with unmodified human cord blood [5, 6] or adult bone marrow (BM)-derived HSPCs [6] demonstrated significantly higher engraftment, with better representation of erythroid [5, 6] and myeloid [5] cells and comparable B and T lymphocyte count when compared to either non-irradiated [5] or irradiated NSG mice [7]. Although the model does not support circulating human red blood cells [5, 6, 8], its developmentally appropriate expression of globin genes and abundant erythroid cells in the BM make it an excellent platform for gene therapy studies targeting hemoglobinopathies.

While the engraftment and hematopoietic potential of unmodified human CD34^+^ cells from cord blood or mobilized peripheral blood (mPB) have been characterized in NBSGW mice and, in some studies, compared with NSG mice [5–7, 9], no direct comparison has assessed gene-modified HSPC engraftment and long-term maintenance in the two models. Here, we evaluated these parameters in the context of sickle cell disease (SCD) gene therapy using mPB CD34^+^ HSPCs modified by either lentiviral vector (LVV) transduction or by CRISPR/Cas9-mediated gene correction with a single-stranded oligodeoxynucleotide (ssODN) donor template to modify the sickle mutation in exon 1 of the human *HBB* gene to the wild-type sequence [10].

## Results and discussion

### Enhanced chimerism in NBSGW mice obscures engraftment deficits of HSPCs harboring high VCN

**Fig. 1:**
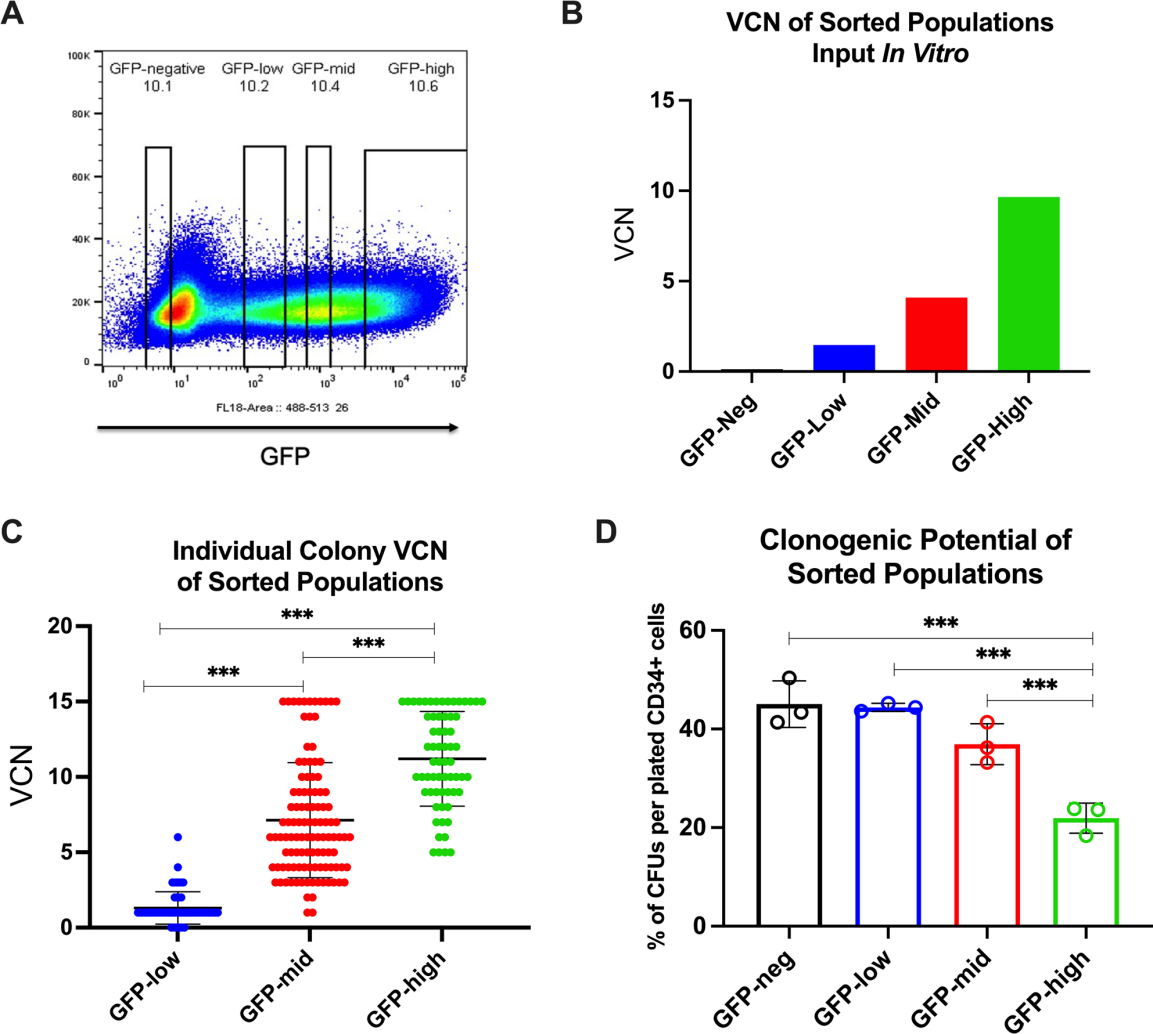
In vitro assessment of hematopoietic potential in LVV-transduced HSPC sub-populations. **A** Sorting strategy to stratify cell populations into low to high VCN populations by transgene expression. **B** VCN of the sorted bulk population assessed by quantitative real-time PCR. **C** VCN quantification performed within individually isolated methylcellulose colonies from sorted populations to assess VCN distribution (GFP-low *n* = 56, GFP-Mid *n* = 110 and GFP-High *n* = 64 colonies evaluated). **D** Analysis of the clonogenic potential of sorted populations at 12–14 days after plating cells in methylcellulose. One sorting experiment with 3 independent replicates plated for CFU analysis. Error bars, mean ± sd. Differences are not significant if not specified: p***< 0.001 by ANOVA followed by pairwise comparisons

To evaluate whether vector copy number (VCN) distribution is influenced by stem cell phenotype, mPB CD34^+^ HSPCs from heathy donors (HD) were transduced with a GFP-expressing LVV (MNDU3-GFP-LVV) [11] and stratified by fluorescence-activated cell sorting (FACS) into GFP-negative, GFP-low, GFP-mid, and GFP-high populations (Fig. 1A), after which VCN was quantified in each group (Fig. 1B). The Colony Forming Unit (CFU) assay, a surrogate for in vivo HSPC repopulation capacity, was used to measure VCN of individual colonies from the three sorted populations (Fig. 1C) and to evaluate the colony-forming potential across different VCN levels (Fig. 1D). Analysis of the hematopoietic potential of the sorted populations revealed a significant reduction in the colony-forming capacity of colonies with high VCN (GFP-high population) (Fig. 1C and D).

**Fig. 2:**
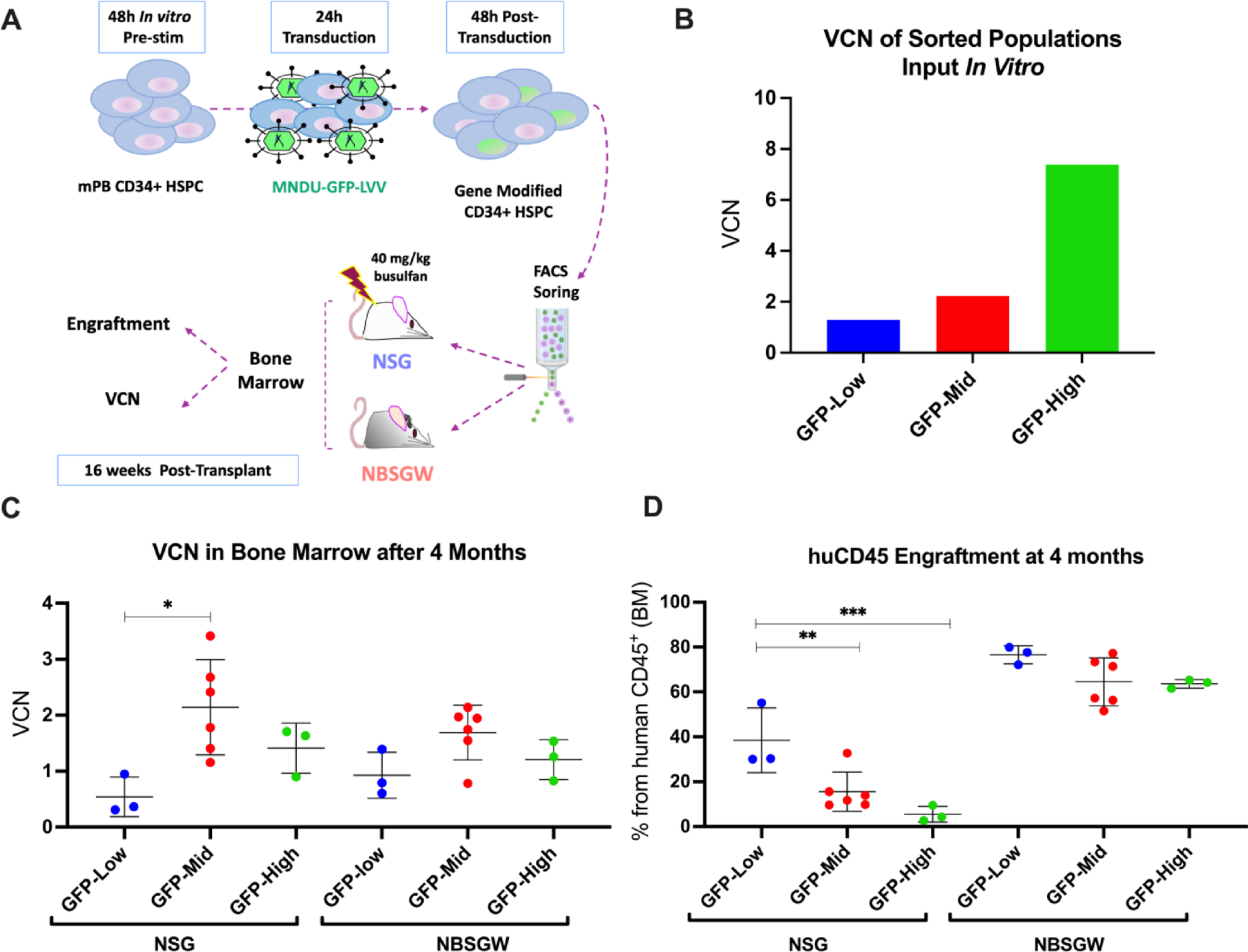
Analysis of engraftment capacity of HSPCs with differing transduction efficiencies in NSG and NBSGW xenotransplant models. **A** Experimental schema for transduction of mPB HSPCs, stratification of cells into low, mid, and high VCN populations by transgene expression, and in vivo transplants. **B** VCN was analyzed by quantitative real-time PCR from the three sorted population in vitro (Input VCN). **C** At 16 weeks post-transplant, VCN was analyzed by quantitative real-time PCR from the BM of mice transplanted with the three sorted populations. **D **Human engraftment in BM assessed by flow cytometry as the percentage of human CD45^+^ cells normalized to the percentage of human CD45^+^ cells plus the percentage of murine CD45^+^ cells. GFP-low *n* = 3 mice, GFP-mid *n* = 6 mice, GFP-High *n* = 3 mice. All mice were 6–8 weeks-old females at the time of the transplant. Error bars, mean ± sd. Differences are not significant if not specified: p*< 0.05, p**< 0.01, p***<0.0001 by ANOVA followed by pairwise comparisons

To further characterize whether transduction efficiency was independent of HSPC phenotype, a second experiment was performed in which sorted populations (GFP-low, GFP-mid, and GFP-high) were transplanted at equivalent cell doses into either NBSGW or busulfan-conditioned NSG mice (Fig. 2A). We observed that the elevated VCN displayed in vitro in the GFP-high group was lost by 16-weeks post-transplant in both mouse models (Fig. 2B and C). In the NSG model, human CD45 engraftment was significantly higher in the GFP-low (38.5%) compared with the GFP-mid (15.5%, *p* < 0.002) and the GFP-high (5.5%, *p* < 0.00002) groups. In contrast, engraftment was overall higher and differences across GFP-low (76.5%), GFP-mid (64.5%) and the GFP-high (63.6%) groups were modest and not statistically significant in the NBSGW model (Fig. 2D). Additionally, as seen in Supplementary Fig. 1 A and 1B, the GFP-high cell population contains significantly fewer phenotypic long-term HSCs and more differentiated progenitor and precursor subpopulations compared to the GFP-negative, GFP-low and GFP-mid populations. Altogether, these results suggest that the HSPC subset most permissive to high levels of transduction is largely distinct from the population with durable long-term engraftment potential. In addition, a high VCN in input cells may contribute to genotoxic stress, as multiple productive vector integrations could impair the capacity of HSPCs to sustain long-term engraftment. However, because only a limited number of unique clones typically engraft in humanized mice, this genotoxic effect cannot be definitively resolved by these models. Notably, although engraftment deficit was not observed in the NBSGW model when assessed by human CD45 + chimerism, this metric reflects the overall frequency of hematopoietic reconstitution but fails to differentiate between true long-term repopulating HSCs and lineage-committed progenitors. This highlights the risk of misinterpreting the safety profile of a given cell product and potentially underestimating genotoxicity when relying solely on this model.

### HSPCs modified by homologous directed repair after CRISPR/Cas9 editing result in comparable gene correction rates in both NSG and NBSGW mouse models 

**Fig. 3:**
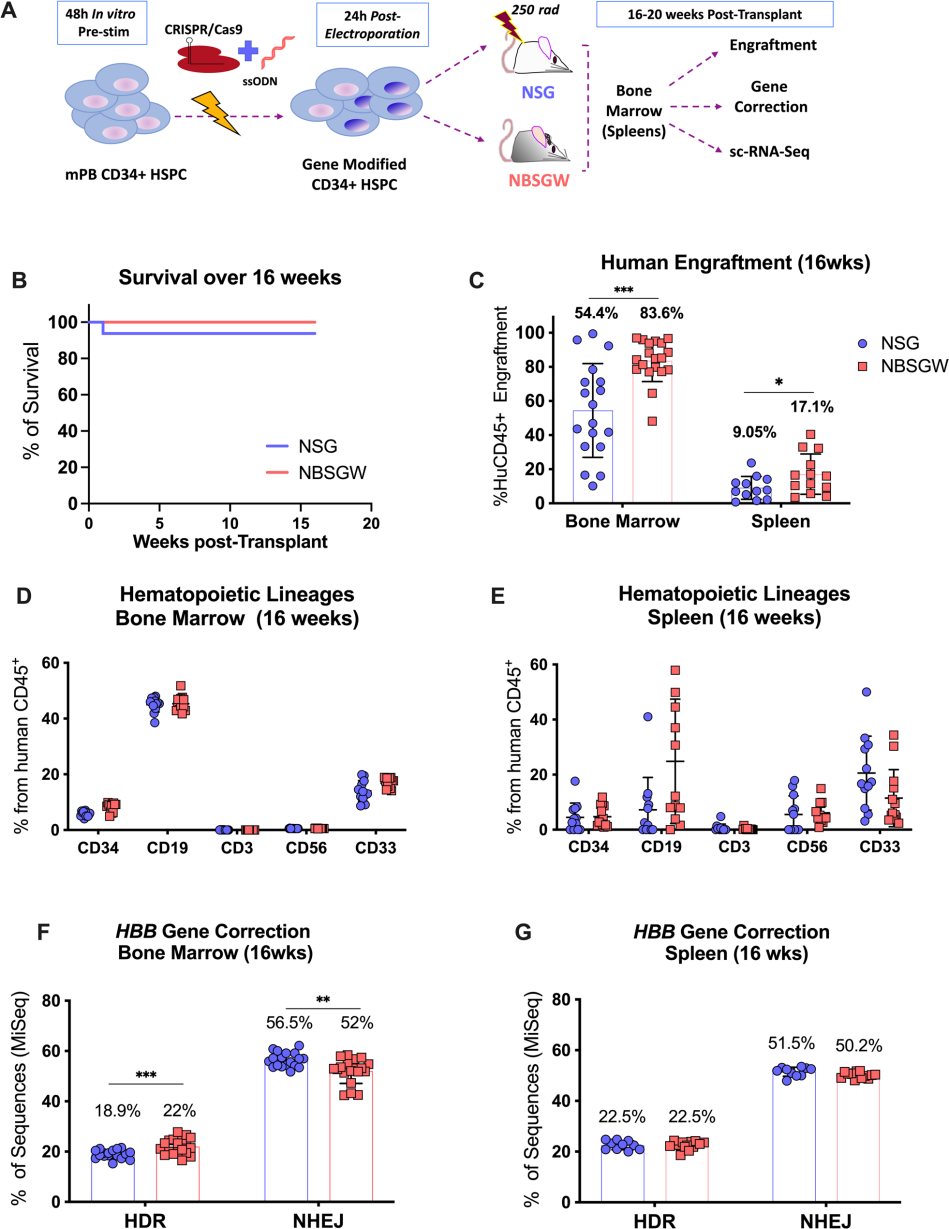
Evaluation of the long-term repopulation capacity of CRISPR/Cas9 edited HSPCs in the NSG and NBSGW mouse models. **A** Experimental schema for homology-directed gene correction at the *HBB* sickle mutation. **B** Survival of mice after transplant. Day 0 (NSG: *n* = 19; NBSGW: *n* = 19), day 112 (NSG: *n* = 18; NBSGW: *n* = 19). **C** Human engraftment assessed 16-weeks post-transplant by flow cytometry in BM and spleen, as the percentage of human CD45^+^ cells normalized to the percentage of human CD45^+^ cells plus the percentage murine CD45^+^ cells. Immunophenotypic analysis was performed 16-weeks post-transplant by flow cytometry in **D** BM; and **E** Spleens. Percentage of HSCs (CD34), B-cells (CD19), T-cells (CD3), NK cells (CD56) and myeloid cells (CD33) from the total human CD45^+^ cells was quantified. **F** BM gene correction (HDR at *HBB*), and allelic disruption (NHEJ at *HBB*) were assessed by next generation sequencing (NGS) in the human CD45^+^ cells enriched from the murine BM 16-weeks post-transplant. **G** Gene correction (HDR at *HBB*), and allelic disruption (NHEJ at *HBB*) were assessed by NGS in the cells isolated from the murine spleens 16-weeks post-transplant. Bone Marrow: NSG *n* = 18, and NBSGW, *n* = 19; Spleens: NSG *n* = 10, and NBSGW *n* = 13; from two independent electroporations and transplants. All mice were 6-week-old females at the time of transplant. Numbers above the bars represent the average value for each condition. Error bars, mean ± sd. Differences are not significant if not specified: p*< 0.05, p**< 0.01, p***<0.0001 by lineal model

CRISPR/Cas9-based gene correction in CD34^+^ HSPCs is limited by multiple sources of cytotoxicity including electroporation stress, generation of double strand breaks (DSB) that trigger the DNA-damage response pathway [12, 13], and the presence of an exogenous DNA donor template [12, 14, 15], all of which reduce the clinical utility of modified cells for downstream applications [16]. While the NSG mouse model has traditionally been used to evaluate the long-term engraftment of HSPCs corrected by homology directed repair (HDR) [10, 12, 13, 15, 17–19], in this study we directly compared the engraftment capacity and long-term maintenance of CRISPR/Cas9-edited mPB CD34^+^ HSPCs [10, 20] in sub-myeloablative irradiated NSG (250 rads) and unconditioned NBSGW mice (Fig. 3A). The CRISPR/Cas9 model used for these investigations targets the *HBB* gene utilizing an ssODN that corrects the sickle mutation to wild-type. Additionally, it alters the PAM sequence to prevent re-cleavage by the endonuclease and introduces a conservative nucleotide substitution adjacent to the SCD mutation site, which serves as a surrogate for SCD correction when using HD CD34^+^ HSPCs [10, 20].

To account for both ssODN- and electroporation-induced acute cytotoxicity, the same number of viable cells was transplanted in both models. Edited mPB CD34^+^ cells with 30.3% HDR and 42.1% non-homologous end joining (NHEJ) were transplanted into both mouse models 24 h post-electroporation. No differences in survival were observed between the two mouse models (Fig. 3B). At 16-weeks post-transplant, human chimerism was significantly lower in NSG mice compared to NBSGW mice (BM: 54.4 ± 27.5% vs. 83.6 ± 12.8%; spleen: 9.0 ± 6.7% vs. 17.1 ± 11.8%) (Fig. 3C). Lineage analysis in BM and spleen revealed no skewing across tissues or mouse strains (Fig. 3D and E). *HBB*-targeted next generation sequencing (NGS) of BM (Fig. 3F) and spleen (Fig. 3G) revealed comparable HDR and NHEJ rates between the two models. In BM, HDR and NHEJ were 18.9 ± 1.7% and 56.5 ± 2.9% in NSG mice, vs. 22.0 ± 3.1% and 52.0 ± 4.9% in NBSGW mice. In the spleen, both models showed nearly identical HDR and NHEJ frequencies (NSG 22.5 ± 1.8% HDR, 51.5 ± 1.8% NHEJ; and NBSGW 22.5 ± 1.6% HDR, 50.2 ± 1.2% NHEJ). Although differences in BM values reached statistical significance, the magnitude of change (e.g., HDR: 19% vs. 22%) may not be biologically meaningful. Finally, in both models we observed that HDR and NHEJ frequencies measured in vitro did not translate proportionally in vivo. This discrepancy likely reflects the limited capacity of long-term reconstituting HSCs to undergo HDR, as well as the restricted repopulation capacity of progenitor cells that repair DSBs through HDR [18]. This phenomenon is inherent to HSC biology rather than a consequence of preferential selection within the mouse models.

Because edited cells were derived from the same input pool and HDR-corrected long-term engrafting hematopoietic stem cells (HSCs) are not expected to have a selective advantage, the frequency of edited and non-edited HSCs should be comparable in both models. However, at the cell dose administered (8e5 cells/mouse), HDR frequencies were significantly higher in the BM of the NBSGW mice compared to the NSG mice (Fig. 3F). In this context, the *c-Kit* mutation of NBSGW mice may allow the engraftment of more committed CD34^+^ progenitor cells, which are less quiescent and therefore more permissive to HDR compared to true long-term HSCs. To explore this possibility, we performed a limiting dilution assay and measured the frequency and gene correction rates of engrafted human cells.

**Fig. 4:**
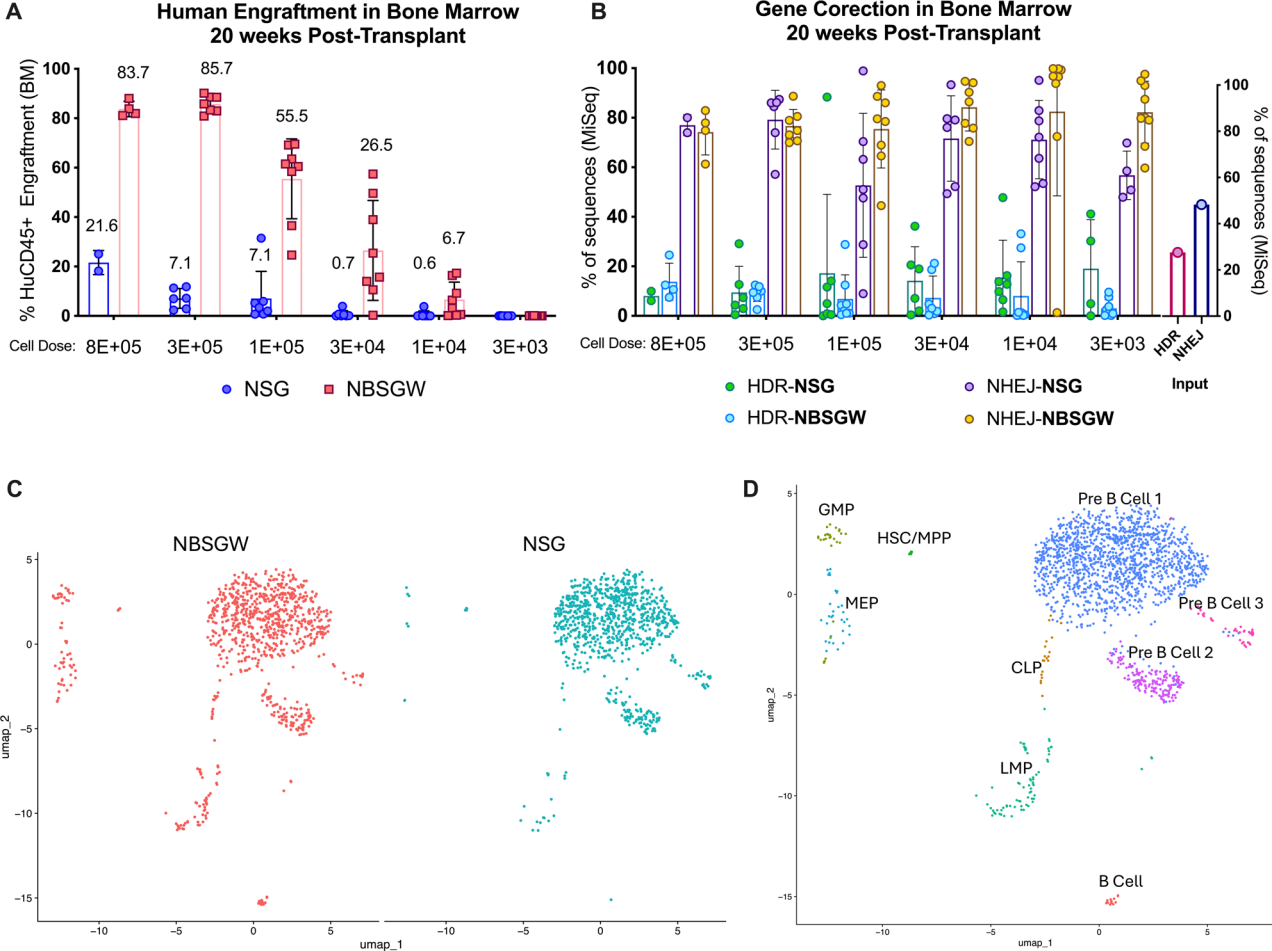
Limiting dilution assay and single-cell RNA-sequing analysis of long-term engrafting HSPCs in the NSG and NBSGW models. For the Limiting Dilution Assay HSPCs were edited in bulk via a single electroporation targeting the sickle mutation. 24 h post-electroporation: 8e5, 3e5, 1e5, 3e4 1e4 and 3e3 cells were transplanted into irradiated NSG recipients (250-rads) and non-conditioned NBSGW mice. Mice were euthanized 20-weeks post-transplant to assess **A **human engraftment in bone marrow (BM) by flow cytometry (numbers above the bars represent the average percentage for each condition); and **B** gene correction (HDR at *HBB*), and allelic disruption (NHEJ at *HBB*) by NGS in the human CD45^+^ cells enriched from the murine BM. Input HDR and NHEJ percentages are displayed at the right of the graph. At the time of harvesting 8e5 (NSG: 2 mice; NBSGW: 4 mice), 3e5 (NSG: 6 mice; NBSGW: 7 mice), 1e5 (NSG: 7 mice; NBSGW: 8 mice), 3e4 (NSG: 7 mice; NBSGW: 8 mice), 1e4 (NSG: 7 mice; NBSGW: 8 mice) and 3e3 (NSG: 8 mice; NBSGW: 9 mice). All mice were 6-week-old females at the time of the transplant. Error bars, mean ± sd. Linear model was used to estimate the effect of cell-dose and mouse model on the outcomes of human engraftment; ANOVA followed by pairwise comparison to investigate differences in HDR and NHEJ. Due to low engraftment rates insufficient reads were recovered in the 3e3 cell dose; therefore, these data were not included in the statistical analysis. Significant differences are described in the main text. Single-cell RNA-sequencing (scRNA-seq) analysis of human CD34^+^ HSPCs enriched from NSG and NBSGW mice 20-weeks post-transplant was performed. **C** UMAP visualization of clustered bone marrow–derived human CD34^+^ HSPC showing cells from NBSGW (left) and NSG (right). For a fair comparison, 908 cells from each mouse model were randomly selected. Each point represents a single cell; and the distribution illustrates differences in hematopoietic lineage reconstitution between the two mouse strains. **D** UMAP of scRNA-seq using the RunUMAP, FindNeighbors, and FindClusters functions for visualization. Clusters were manually annotated using the top 50 differentially expressed genes using FindAllMarkers. Hematopoietic Stem Cell (HSC), multipotent progenitors (MPP), Lympho-Myeloid Progenitor (LMP), Common Lymphoid Progenitor (CLP), Granulocyte Monocyte Progenitor (GMP), and Megakaryocytic-Erythroid Progenitor (MEP)

In this set of transplants, edited mPB CD34^+^ HSPCs (input cells: 27.6% HDR, 48.3% NHEJ) were injected into sub-myeloablative irradiated NSG (250 rads) or unconditioned NBSGW mice at equivalent doses (8e5, 3e5, 1e5, 3e4, 1e4, and 3e3 cells per mouse). Twenty weeks post-transplant, NBSGW mice demonstrated robust engraftment at the 8e5 and 3e5 doses (83.7 ± 3.0% and 85.7 ± 3.4%, respectively) with stepwise declines at lower doses (55.5 ± 16.2% at 1e5; 26.5 ± 20.2% at 3e4; 6.7 ± 7.0% at 1e4; and undetectable at 3e3; Fig. 4A). In contrast, the NSG group exhibited more limited engraftment (21.6 ± 4.9% at 8e5; 7.1 ± 4.0% at 3e5; 7.1 ± 10.9% at 1e5; and < 1% at 3e4 and 1e4; and undetectable at 3e3; Fig. 4A). In all, these experiments demonstrate that the NBSGW model showed significant higher human engraftment than the NSG model excepting at the two lowest doses (1e4 and 3e3); and that the NSG mice require at least 1e5 edited-cells to allow measurable engraftment in the BM after 20 weeks while the NBSGW model needs ten times fewer edited-cells to achieve measurable engraftment in at least 50% of mice.

NGS analysis of *HBB* in BM samples revealed no significant differences in the average frequencies of HDR or NHEJ between the two mouse models (with the exception of the NHEJ rate at the 1e5 cell dose), nor within each model across the range of input cell doses (Fig. 4B). The discrepancy between results at the 8e5 cell dose in Fig. 3F vs. Fig. 4B likely reflects the smaller sample size in Fig. 4B (*n* = 2–4) at that cell dose. In contrast, analysis of the less saturating, decreasing input doses (*n* = 6–9 mice per dose and model) showed no differences in gene correction outcomes between mouse models at any dose, confirming that the NBSGW model does not selectively enhance the maintenance of more committed CD34^+^ progenitor cells relative to the NSG model.

### Single-cell RNA-sequencing analysis identifies distinct hematopoietic sub-populations under-represented in the NSG model

Evaluation of long-term engraftment in humanized mouse models typically relies on measuring human CD45^+^ chimerism, which reflects the overall frequency of hematopoietic reconstitution but does not differentiate between true long-term repopulating HSCs and lineage-committed progenitors. While lineage analyses (Fig. 3D and E) provide additional resolution, they remain insufficient to define HSC properties. To address this limitation, we applied single-cell RNA-sequencing (scRNA-seq) to profile human CD34^+^ enriched HSPCs isolated from the BM of both models.

Due to the significantly higher levels of human engraftment in the NBSGW model, a greater number of human CD34^+^ enriched cells were recovered by FACS from the NBSGW compared to NSG mice for scRNA-seq. To account for this imbalance and enable fair comparison, we applied down-sampling to randomly selected cells (*n* = 908) from each model for analysis (Fig. 4C). Manual cluster annotation was performed using the top 50 differentially expressed genes identified by *FindAllMarkers*, (Fig. 4D) with reference to a previously published cell type marker list [21–24] (Supplementary Table 1).

Populations clustering as granulocyte monocyte progenitors (GMP) and megakaryocytic-erythroid progenitors (MEP) were observed in the NBSGW model but nearly absent in NSG mice. Similarly, clusters corresponding to lymphoid-myeloid progenitors (LMP) and common lymphoid progenitors (CLP) were well represented in NBSGW mice yet markedly underrepresented in NSG recipients (Fig. 4C–D and Supplementary Fig. 2). The presence of these progenitor populations in NBSGW mice suggests a greater capacity to sustain human hematopoiesis from long-term engrafting HSCs, resulting in a larger overall fraction of human cells and therefore explaining the higher human CD45^+^ chimerism observed in this model. Notably, the relative enrichment of MEPs in NBSGW is consistent with their ability to support human erythropoiesis and platelet formation compared to irradiated NSG recipients [25]. In contrast, clusters corresponding to Pre B Cell 1, 2 and 3 were strongly represented in both models, reflecting that B cells constitute the predominant human population produced in these systems [5]. Finally, a small cluster corresponding to HSCs/multipotent progenitors (MPP) was identified with comparable representation in both models (Fig. 4C–D and Supplementary Fig. 2).

## Conclusions

In LVV-modified HSPCs, increasing VCN was associated with impaired long-term engraftment in NSG mice. This engraftment deficit was not evident in the NBSGW model, where higher overall human chimerism may obscure the detection of cells with reduced repopulating potential.

In contrast, HSPCs modified by HDR with CRISPR/Cas9 and a corrective ssODN exhibited comparable gene correction rates in both models at equivalent input doses 20-weeks post-transplant, indicating that both models have the same capacity to engraft long-term repopulating HSCs. However, scRNA-seq identified four distinct populations classified as LMP, CLP, GMP and MEP that were markedly under-represented in the NSG model. The presence of these populations underscores the greater capacity of the NBSGW model to support multilineage human hematopoiesis, consistent with the higher overall proportion of human CD45^+^ cells detected by flow cytometry.

Collectively, these results establish that both the NBSGW and NSG murine models are suitable for assessing gene modifications in long-term engrafting HSCs. However, the enhanced chimerism observed in NBSGW mice, as measured by human CD45%, may obscure intrinsic properties of the input product that negatively affect engraftment, such as a high proportion of cells with high VCN that are unlikely long-term repopulating HSCs. Furthermore, any impact of these high VCN cells on overall engraftment could be exacerbated by genotoxic stress, including replication-induced genome instability [26], potentially leading to premature graft exhaustion. This limitation does not appear to apply to site-specific gene editing, in which modifications are theoretically occurring at a single genomic locus. In this context, genotoxicity associated with site-specific gene editing is tightly linked to the off-target profile of the therapeutic gRNA. Unintended DSB at off-target sites can lead to large deletions, chromosomal rearrangements, or translocations, resulting in genomic instability. This risk has been substantially reduced through the use of high-fidelity nucleases and optimized gRNAs. In the present study, the gRNA being used has a highly restricted off-target profile, with only a single predominant off-target site identified and no detectable translocations [10, 20]. Taken together, these findings underscore that interpretation of engraftment and safety outcomes is highly dependent on the biological context and model system employed. Careful selection and understanding of in vivo models are therefore essential for accurate preclinical assessment of gene therapy products prior to clinical translation.

## Supplementary Information

Supplementary Material 1.

Supplementary Material 2.

## Data Availability

The NGS datasets generated and/or analyzed during the current study have been deposited in the SRA under BioProject ID PRJNA1337400. https://www.ncbi.nlm.nih.gov/bioproject/PRJNA1337400. The scRNA-seq datasets generated and/or analyzed during the current study are available in the Gene Expression Omnibus (GEO) repository (accession number # GSE303930). https://www.ncbi.nlm.nih.gov/geo/query/acc.cgi?acc=GSE303930.

## Abbreviations

HSPCs: Hematopoietic stem and progenitor cells
LVV: Lentiviral vector
HSCs: Hematopoietic stem cells
BM: Bone Marrow
SCD: Sickle cell disease
ssODN: Single-stranded oligonucleotide
VCN: Vector copy number
mPB: Mobilized peripheral blood-derived
HD: Heathy donors
FACS: Fluorescence-activated cell sorting
HDR: Homology directed repair
NHEJ: Non-homologous end joining
NGS: Next generation sequencing
scRNA-seq: Single-cell RNA-sequencing
GMP: Granulocyte monocyte progenitors
MEP: Megakaryocytic-erythroid progenitors
LMP: Lymphoid-myeloid progenitors
CLP: Common lymphoid progenitors
MPP: Multipotent progenitors
