# Supplementary material for: Comparative analysis of NSG and NBSGW mice for preclinical evaluation of gene-modified human hematopoietic stem and progenitor cells
Source: Stem Cell Res Ther. 2026 Apr 15;17:196. doi: 10.1186/s13287-026-05010-8 (PMC13196111; doi:10.1186/s13287-026-05010-8)
Supplement: Supplementary file 2 — Supplementary Material 2. [file 13287_2026_5010_MOESM2_ESM.docx]

**Supplementary Material**

**Figures**

**
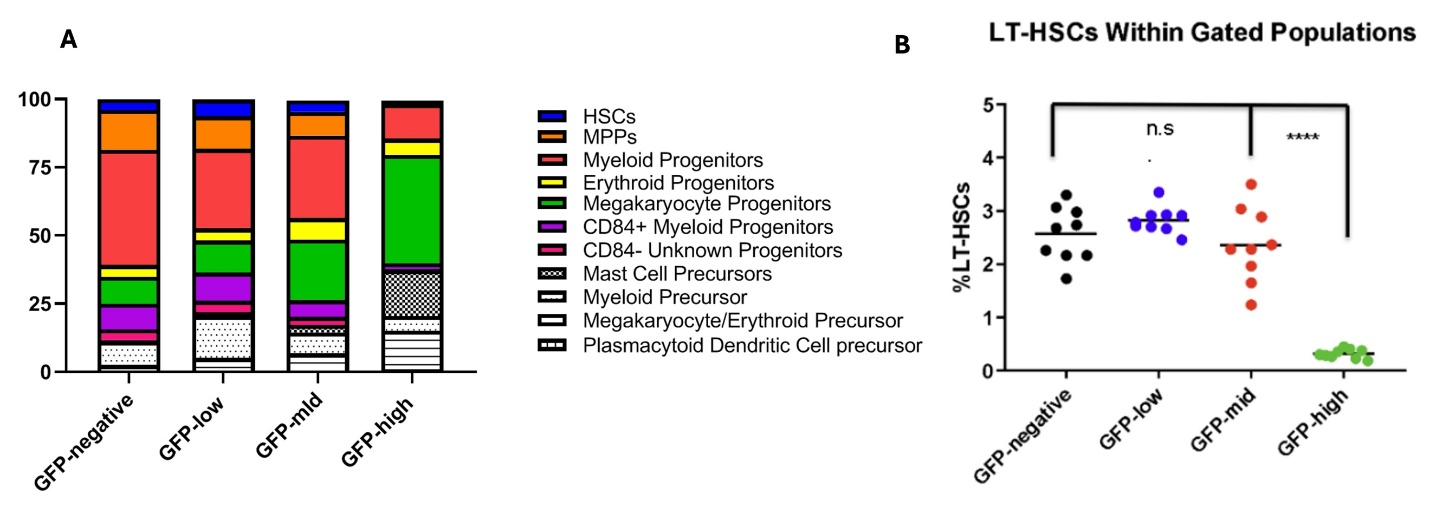
**

**Supplementary Figure 1. Phenotypic characterization and transduction potential of the input GFP+ cell subpopulations.** (**A**) CyTOF immunophenotyping was performed on the sorted GFP populations (GFP-negative, GFP-low, GFP-mid, GFP-high) and ViSNE analysis was performed to allow for high dimensional protein expression profiling of CD34+ subpopulations on a single cell basis. Compared to the GFP-negative, GFP-low and GFP-mid sorted populations, the GFP-high sorted population had reduced levels of HSCs and MPPs and an enrichment of more differentiated progenitor and precursor subpopulations. (**B**) Phenotypically defined long-term HSCs (CD34+/CD38low/CD45RA-/CD90+) were FACS-isolated, labeled with carboxyfluorescein succinimidyl ester (CFSE), recombined with bulk CD34+ cells and transduced with a GFP LVV. GFP fractions were then analyzed for proportion of long-term HSCs contained within each GFP subset. Compared to the GFP-negative, GFP-low and GFP-mid populations, the GFP-high population contained significantly fewer phenotypic long-term HSCs.


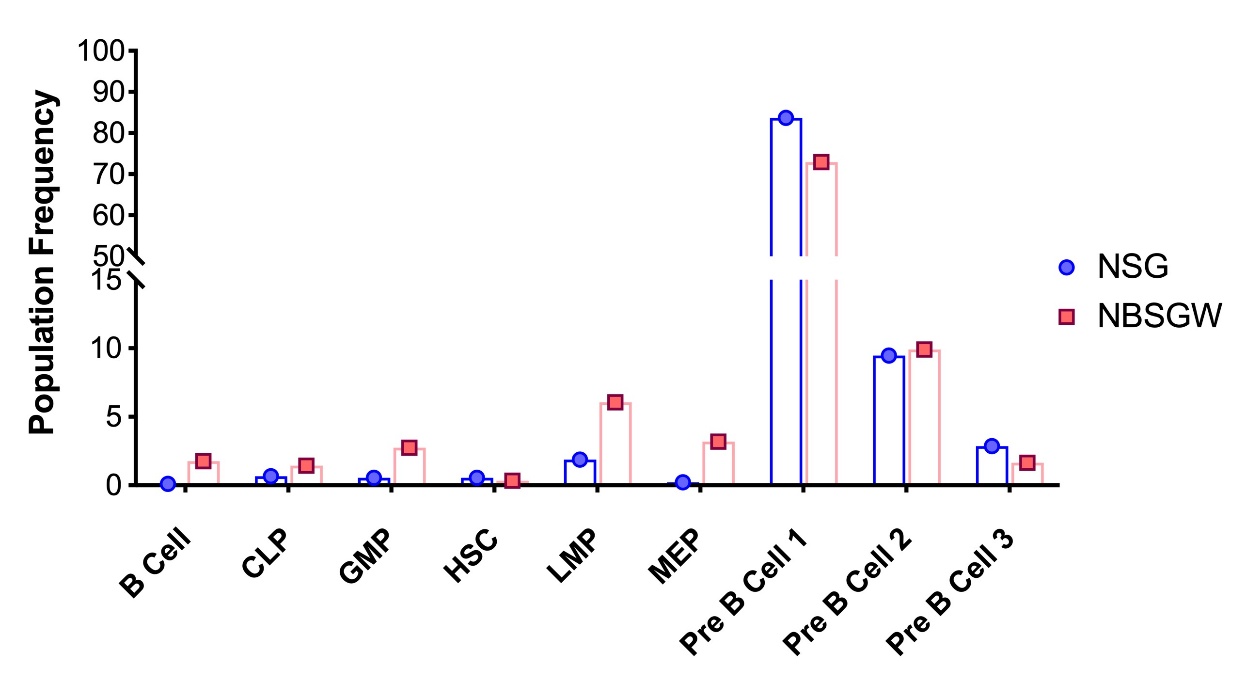


**Supplementary Figure 2. Hematopoietic subpopulation frequencies in NSG vs. NBSGW mouse models.**

**Methods**

**Cell count and viability**

Cell viability and cell numbers were determined by manual counting using Trypan Blue (Sigma, Cat. T8154) staining, disposable hemacytometers (INCYTO, Cat. DHC-N01-5), and an inverted microscope.

**CD34^+^ isolation, pre-stimulation, lentiviral transduction, sorting, and *in vitro* culture**

G-CSF mobilized apheresis products from healthy donors were obtained from CGT Global (Folsom, CA) enriched using magnetic CD34 beads (Miltenyi, Germany) and cryopreserved. CD34^+^ cells were thawed and pre-stimulated for 48h at 1e6 cells/ml using CellGro stem cell growth media (SCGM; CellGenix, Freiburg, Germany), supplemented with recombinant human cytokines stem cell factor (SCF), fms-related tyrosine kinase 3 ligand (Flt3-L), and thrombopoietin (TPO) at 100 ng/ml and Interleukin 3 (IL3) at 20 ng/ml (CellGenix, Freiburg, Germany). Cells were transduced with a research grade GFP-expressing lentiviral vector (Genetix Biotherapeutics,, Somerville, MA) in cytokine-supplemented media for 24h as protamine sulfate at a final concentration of 8 μg/ml (APP Pharmaceuticals, Schaumburg, IL) for 24h at 4e6 cells/ml to target a transduction efficiency of approximately 50% by GFP expression. Cells were then washed and maintained in cytokine-supplemented media prior to being subjected to sorting by GFP expression after 48h of transduction using either a FACS Aria (BD Biosciences) or MoFlo (Beckman Coulter). Gates were set to target ~10% of the live cell population for GFP negative cells (GFP neg), 10% of the lowest GFP expressing cell population (GFP low), 10% of the median of GFP expressing cells (GFP mid), and 10% of the brightest GFP expressing cells (GFP high). Sorted cells were subsequently either put into *in vitro* assays or transplanted into immunodeficient mice.

For methylcellulose colony-forming unit (CFU) assays following lentiviral transduction, approximately 500 cells in a 300ul volume of SCGM were plated into a single 3ml tube of MethoCult Classic H4434 (StemCell Technologies, Vancouver, BC), and approximately 1.1ml was dispensed into a well of a 6-well SmartDish (StemCell Technologies, Vancouver, BC). Following 12 to 16 days of culture, colonies were scored by morphology, enumerated, and either plucked as individual colonies or pooled and subjected to quantitative PCR (qPCR) for assessment of vector copy number (VCN).

**Vector copy number assessment**

Genomic DNA (gDNA) was isolated from cell cultures using QIAGEN DNeasy protocol (QIAGEN, Hilden, Germany). qPCR was performed using TaqMan Fast Master Mix (Invitrogen, Carlsbad, CA, USA) and 0.9 μM GAG forward (5′-GGAGCTAGAACGATTCGCAGTTA-3′) and reverse (5′-GGTTGTAGCTGTCCCAGTATTTGTC-3′) primers and GAG FAM probe [5′-(FAM)-ACAGCCTTCTGATGTCTCTAAAAGGCCAGG-(TAMRA)-3′] and RNASE-P-VIC control TaqMan assay (Invitrogen), and run using Fast program on Applied Biosystems StepOnePlus real-time thermocycler (Invitrogen). VCN was assessed relative to an internal reference clone, Clone K3 cDNA, known to contain two copies of integrated viral DNA per diploid genome.

From the CFU assay, individual colonies were plucked from methylcellulose into lysis buffer, and stabilization buffer was added per manufacturer’s protocol (TaqMan Sample-to-SNP, Thermo Fisher Scientific, Waltham, MA). gDNA from colony lysates was analyzed via qPCR to determine VCN.

**Xenotransplantation of lentiviral-transduced CD34^+^ HSPCs**

All work with mice was done under protocols approved by a local institutional animal care and use committee (IACUC; bluebird bio (now known as Genetix Biotherapeutics)).

Female, 6 to 8-week-old NSG or NBSGW mice (The Jackson Laboratory, Bar Harbor, ME) were transplanted intravenously with 3e5 CD34^+^ HSPC sorted by GFP expression. Only the NSG mice were conditioned with 40 mg/kg busulfan one-day prior to transplantation. Mice were maintained in sterile conditions and provided with food and water *ad libitum*. 16-weeks post-transplant, bone marrow (BM) from the femurs were collected to assess human engraftment determined by flow cytometry and calculated as (huCD45^+^/ [huCD45^+^ + muCD45^+^]) x100. Additionally, BM was processed for gDNA and VCN analysis. Antibodies were purchased from BioLegend and BD Bioscience.

All the animal work has been reported in line with the ARRIVE guidelines 2.0

**CD34^+^ isolation, pre-stimulation and gene correction by electroporation**

Plerixafor and G-CSF mobilized peripheral blood leukopaks were purchased from Charles Rivers (Van Nuys, CA) and automated CD34^+^ isolation using the CliniMACS Plus (Miltenyi Biotec, Gaithersburg, MD) was performed. CD34^+^ cells were frozen by the control rate freezer on CryoStor CS5 (STEMCELL Technologies, Vancouver, BC, Canada) and cryopreserved CD34^+^ cells were stored in the vapor phase of liquid nitrogen (LN2) for further use.

Isolated CD34^+^ cells were thawed and pelleted by centrifugation followed by resuspension in culture medium. Cells were cultured in X-VIVO-15 serum-free medium (Lonza, Basel, Switzerland) with PSG (penicillin, streptomycin, glutamine [Gemini Bio-Products, Sacramento, CA]), and recombinant human cytokines: SCF, Flt3-L, TPO; all at 50ng/ml (Peprotech, Rocky Hill, NJ), for a pre-stimulation phase of ~48h at 5e5 c/ml. Prior to electroporation, cells were pelleted (10 min at 200xg) and resuspended in Hyclone Maxcyte electroporation buffer (Cytiva, Cat # EPB-1) to be mixed with gene editing reagents [3.16 µM sgRNA (Synthego, Cat. 3xMS-G10), 2.74 µM SpyFi high-fidelity Cas9 protein (Aldevron, Cat. 9216), and 3.18 µM ssDNA CJ6A (TriLink, Cat. T018). CD34^+^ cells were electroporated using the MaxCyte GT (Maxctye, Rockville, MD) in HyClone MaxCyte Electroporation Buffer, with instrument set to power level 7, and put back in culture in pre-stimulation media at 5e5c/ml. 24h post-electroporation cells were harvested, and viability was assessed by trypan blue exclusion. For mice transplants the corresponding cell number needed for each assay was harvested at this time point. The remaining CD34^+^ cells were maintained in myeloid expansion medium (Iscove’s Modified Dulbecco’s Medium [IMDM, ThermoFisher Scientific, Waltham, MA] + 20% FBS [HI fetal bovine serum (FBS), Gibco/Thermo-Fisher, Waltham, MA] + 0.52% Bovine Serum Albumin (BSA, Sigma Aldrich, St. Louis, MO) + 5 ng/ml IL3, 10 ng/ml Interleukin 6 [IL6], 25 ng/ml SCF [Peprotech, Rocky Hill, NJ]), for 5 additional days, after which gDNA isolation was performed (PureLink Genomic DNA Mini Kit, Invitrogen/Thermo Fisher Scientific, Carlsbad, CA) for subsequent next generation sequencing (NGS) to assess gene correction and allelic disruption rates.

**Genotyping by NGS**

The target site in *HBB* was amplified from gDNA by polymerase chain reaction (PCR) according to standardized methodology. Briefly, the amplicons were pooled and loaded on an Illumina MiSeq instrument at the Berkeley Genome Sequencing Laboratory and sequenced with a 2x300 paired end read kit (Illumina MiSeq V3), 10-30% PhiX spike-in, and manufacturer’s loading instructions. Resulting FASTQ files were analyzed for genome editing outcomes using a reimplementation of a published procedure [Cortado, a reimplementation of CRISpresso (1)] according to written procedures.

**Xenotransplantation:**

All work with mice was done under protocols approved by the UCLA Institutional Animal Care and Use Committee (IACUC)(animal research committee (ARC) protocol number 2008-167) or the bluebird bio (now known as Genetix Biotherapeutics) IACUC (protocol number 00214).

All mice transplants were performed on 6-week-old female NSG or NBSGW mice (The Jackson Laboratory, Bar Harbor, ME). 24h post-electroporation, transplants were performed 3-5h after 250-cGy total body irradiation (only performed on the NSG mice). Mice were anesthetized with isoflurane via an induction chamber at 3% for induction and 2% for maintenance, using 1.5 L/min oxygen flow. Anesthesia depth was confirmed by the loss of the righting reflex, after which retro-orbital (or intravenous injections) were performed. Animals were monitored in home cages until full recovery. Retro-orbital injections were performed in 80-100 ul of PBS. 8e5 cells were used as standard dose for the first transplant (Figure 3). For the limiting dilutions assay (Figure 4), the following cell numbers were used per mouse: 8e5 (starting number of mice: 4 per strain), 3e5, 1e5, 3e4, 1e4 (starting number of mice: 8 per strain) and 3e3 (starting number of mice: 9 per strain) into irradiated NSG recipients (250 rads) and non-conditioned NBSGW mice.

Mice were euthanized 16-20 weeks post-transplant using a cage lid style CD2 Flowmeter, with CO_2_ administered at a flow rate of 5 liters per minute. Following the last observed breath, the CO_2_ flow was maintained for at least one minute. After turning off the CO_2_ and removing the euthanasia lid, mice were removed for organ harvesting. Two femurs were collected and flushed with MACS buffer. Spleens were squeezed through a 70 μm filter. Red blood cells were lysed using the lysing buffer (BD Biosciences, Franklin Lakes, NJ). The levels of human engraftment were determined on the Fortessa flow cytometer (BD Biosciences) and calculated as (huCD45^+^/ [huCD45^+^ + muCD45^+^]) x100. Lineage distribution was measured by flow cytometry using cell-type specific antibodies: anti-human CD45 (BD Biosciences, Cat. No. 560367), anti-mouse CD45 (Biolegend, [San Diego, CA] Cat. No. 103107), anti-human CD34 (Biolegend, Cat. No. 343608), anti-human CD19 (Biolegend, Cat. No. 302216), anti-human CD56 (BD Biosciences, Cat. No. 555516), anti-human CD3 (Biolegend, Cat. No. 344818), and anti-human CD33(Biolegend, Cat. No. 303424). The human cells from mouse BM were enriched using magnetic CD45 beads microbeads (Miltenyi Biotec**,** Gaithersburg, MD) for subsequent gDNA isolation from BM, and spleens; and the levels of gene editing in human cells were determined by NGS, as described above. Mouse harvest, flow cytometry and sequencing analyses were performed in a blinded manner.

All the animal work has been reported in line with the ARRIVE guidelines 2.0

**Single-cell RNA-sequencing:**

20 weeks post- editing and transplant, two femurs were collected per mouse and flushed with MACS buffer. Murine CD45^+^ cell depletion was performed on the BM harvested from NSG and NBSGW mice. Murine depleted cell-samples from 5-7 mice were pooled per mouse’s strain. To maximize the resolution of more primitive populations cell sorting was performed (Aria H Cell Sorter, BD) on the pooled samples for human lineage depletion (CD19^+^, CD3^+^ and CD33^+^ cells) and human CD34^+^ cell enrichment. Three independent pooled samples (5-7 mice/pool) were sorted per each mouse strain resulting in a total of 3 NSG and 3 NBSGW samples used for single-cell RNA-sequencing (scRNA-Seq). scRNA libraries were prepped using Chromium Single Cell 3’ Gene Expression reagent kit (10x Genomics, Pleasanton, CA) according to the manufacturer’s protocol, to achieve the desired cell recovery target of 10,000 CD34^+^ cells for NBSGW mice, and 1,000 CD34^+^ for NSG mice; and sequenced on Novaseq XPlus with paired-end reads and average sequencing depth of 20,000 read pairs per cell.

Raw sequenced single-cell data was aligned by Cellranger (v7.2) using the human reference genome (hg38) to generate counts matrices and analyzed using the standard Seurat (v5) pipeline (2). For quality control, cells were retained if they met the following criteria: (i) number of detected genes greater than the 1st percentile of the distribution, (ii) total UMI counts between the 1st and 99th percentiles, with a minimum threshold of 1,000 UMIs if applicable, and (iii) less than 10% mitochondrial reads. Data was normalized using default NormalizeData function parameters, FindVariableFeatures to select 2,000 genes with the highest standardized variance and ScaleData to perform z-score transformation. To analyze all samples, we combined the two strains and performed integration using IntegrateData, with Harmony integration (3). The combined data was reanalyzed using the RunUMAP, FindNeighbors, and FindClusters functions for visualization. For a balanced comparison, we randomly selected 908 cells (downsampling) from each strain to perform differential expression between NSG and NBSGW cells (4). We manually annotated the clusters using the top 50 differentially expressed genes using FindAllMarkers.

**Statistical analysis**

ANOVA (analysis of variance) followed by pairwise comparisons were used to estimate difference in (i)VCN of individual colony-forming cells from the three-sorted populations (GFP-low, -mid and -high) (Fig.1C); (ii) the colony-forming capacity of cells with different VCN from the three sorted populations and negative cells (GFP-low, -mid and -high) (Fig 1D); (iii) VCN and human CD45-engraftment in NSG or NBSGW mice transplanted with CD34^+^ HSPCs from the three-sorted populations (GFP-low, -mid and -high) (Fig2 C and D respectively); and (iv) HDR and NHEJ at different cell doses within each mouse model and between the two strains (Fig 4B).

Linear model was used to evaluate the difference in engraftment, HDR and NHEJ between NSG and NBSGW mouse models at bone marrow and spleen (Fig 3C, 3F and 3G); and to estimate the effect of cell-dose and mouse model on human CD45 engraftment outcomes. Interaction effect between cell-dose and mouse model were included in the linear model and appropriate contrasts were used to evaluate the impact of mouse model at different cell-doses and the impact of cell-doses within each mouse model (Fig 4A).

All values are given in the text as mean (±SD) and the p value accepted as significant is stated in each figure.

**References**

1. Pinello L, Canver MC, Hoban MD, Orkin SH, Kohn DB, Bauer DE, et al. Analyzing CRISPR genome-editing experiments with CRISPResso. Vol. 34, Nature Biotechnology. Nature Publishing Group; 2016. p. 695–7.

2. Hao Y, Hao S, Andersen-Nissen E, Mauck WM, Zheng S, Butler A, et al. Integrated analysis of multimodal single-cell data. Cell. 2021 Jun 24;184(13):3573-3587.e29.

3. Korsunsky I, Millard N, Fan J, Slowikowski K, Zhang F, Wei K, et al. Fast, sensitive and accurate integration of single-cell data with Harmony. Nat Methods. 2019 Dec 1;16(12):1289–96.

4. Luecken MD, Theis FJ. Current best practices in single‐cell RNA‐seq analysis: a tutorial. Mol Syst Biol. 2019 Jun;15(6).
